# Dose modification factors for ^192^Ir high‐dose‐rate irradiation using Monte Carlo simulations

**DOI:** 10.1120/jacmp.v7i3.2293

**Published:** 2006-08-24

**Authors:** Bassel Kassas, Firas Mourtada, John L. Horton, Richard G. Lane, Thomas A. Buchholz, Eric A. Strom

**Affiliations:** ^1^ Radiation Oncology Department Greater Baltimore Medical Center Baltimore Maryland 21204 U.S.A.; ^2^ Radiation Physics Department The University of Texas M. D. Anderson Cancer Center Houston Texas 77030 U.S.A.; ^3^ Radiation Oncology Division The University of Texas M. D. Anderson Cancer Center Houston Texas 77030 U.S.A.

**Keywords:** Monte Carlo, MammoSite, HDR, brachytherapy, partial breast irradiation

## Abstract

A recently introduced brachytherapy system for partial breast irradiation, MammoSite, consists of a balloon applicator filled with contrast solution and a catheter for insertion of an I192r high‐dose‐rate (HDR) source. In using this system, the treatment dose is typically prescribed to be delivered 1 cm from the balloon's surface. Most treatment‐planning systems currently in use for brachytherapy procedures use water‐based dosimetry with no correction for heterogeneity. Therefore, these systems assume that full scatter exists regardless of the amount of tissue beyond the prescription line. This assumption might not be a reasonable one, especially when the tissue beyond the prescription line is thin. In such a case, the resulting limited scatter could cause an underdose to be delivered along the prescription line. We used Monte Carlo simulations to investigate how the thickness of the tissue between the surface of the balloon and the skin or lung affected the treatment dose delivery. Calculations were based on a spherical water phantom with a diameter of 30 cm and balloons with diameters of 4 cm, 5 cm, and 6 cm. The dose modification factor is defined as the ratio of the dose rate at the typical prescription distance of 1 cm from the balloon's surface with full scatter obtained using the water phantom to the dose rate with a finite tissue thickness (from 0 cm to 10 cm) beyond the prescription line. The dose modification factor was found to be dependent on the balloon diameter and was 1.098 for the 4‐cm balloon and 1.132 for the 6‐cm balloon with no tissue beyond the prescription distance at the breast–skin interface. The dose modification factor at the breast–lung interface was 1.067 for the 4‐cm balloon and 1.096 for the 6‐cm balloon. Even 5 cm of tissue beyond the prescription distance could not result in full scatter. Thus, we found that considering the effect of diminished scatter is important to accurate dosimetry. Not accounting for the dose modification factor may result in delivering a lower dose than is prescribed.

PACS number: 87.53.Jw

## I. INTRODUCTION

A new high‐dose‐rate (HDR) brachytherapy system, the MammoSite Radiation Therapy System (Proxima Therapeutics Inc., Alpharetta, GA), has been introduced recently to deliver partial breast irradiation after a lumpectomy for early‐stage breast cancer.^(^ ^1^ ^–^ ^5^ ^)^ The system is designed to treat the tissue immediately surrounding the lumpectomy cavity by inserting a multilumen balloon catheter into the cavity at the time of the surgery or shortly thereafter. The balloon, which is currently available in two sizes (4 cm to 5 cm and 5 cm to 6 cm), is then inflated with a contrast solution. This causes the tissue to shape into an approximately spherical shell conforming to the balloon's surface. The HDR source is then loaded into the center of the balloon for a specified duration to deliver the prescribed dose fraction.

A typical treatment prescription is 34 Gy delivered in fractions of 3.4 Gy twice per day for 5 days with a minimum of 6 h between fractions. The dose typically is prescribed to be delivered 1 cm from the balloon's surface. This prescription line introduces a restriction on the distance from the balloon's surface to the skin to limit the dose delivered to the skin and avoid the probability of a poor cosmetic outcome. Therefore, a distance of 5 mm to 7 mm between the skin and the balloon's surface is commonly used as a minimum for such treatments.^(5)^

One shortcoming of current brachytherapy treatment‐planning systems is that they use water‐based dosimetry with no correction for heterogeneity. These systems assume that full scatter exists in clinical applications regardless of the amount of tissue beyond the prescription line. However, this assumption might not be a reasonable one, especially when the tissue beyond the prescription line is thin. In such a case, we believed that the resulting limited scatter could cause an underdose to be delivered along the prescription line. To investigate this possibility, the lack of full scatter resulting from the limited thickness of the tissue between the balloon's surface and the breast's interface with skin or lung tissue was modeled using Monte Carlo simulations for a range of balloon diameters and tissue thicknesses.

## II. MATERIALS AND METHODS

The modeling in this study was based on the techniques used in a previously reported investigation of the effects of contrast on dosimetry in this procedure.^(6)^ The phantom was a sphere with a diameter of 30 cm, and the balloon was assumed to be a sphere positioned at the center of the phantom. Three balloon diameters were simulated (4 cm, 5 cm, and 6 cm) to model all potential clinical applications covered by the manufacturer's recommendations. The effects of the silicone balloon wall and nylon catheter were assumed to be negligible. The material inside the balloon was assumed to be water (11.2% H and 88.8% O by weight). The breast tissue outside the balloon was also modeled as water. In the case of the skin interface, the material beyond the breast tissue was assumed to be dry air (75.53% N, 23.18% O, 1.28% Ar, and 0.0124% C by weight).^(7)^ In the case of the lung interface, the material beyond the breast tissue was assumed to be the lung composition reported by the Medical Internal Radiation Dose Committee,^(8)^ limited to 12 elements (H, C, N, O, Mg, P, S, Ca, Cl, K, Na, and Fe) with a density of 0.2958 g cm^–3^. Other elements collectively account for less than 0.005% of lung tissue by weight and thus were not included in our simulations. For the full scatter simulations, all the phantom material within the 30‐cm sphere was assumed to be water. The dose modification factor is defined here as the ratio of the dose rate 1 cm from the balloon's surface with full scatter to the dose rate with a finite tissue thickness beyond the prescription line.

The source simulated in this study was the MicroSelectron HDR I192r source (v2, model no. 105.002; Nucletron B.V., Veenendaal, Netherlands), which has been described in detail by Daskalov et al.^(9)^ The modeled source is a 100% solid iridium metal cylinder, 3.6 mm in length and 0.65 mm in diameter with beveled edges. The beveled edges of the solid iridium cylinder were not modeled in this simulation. The effect of not including the rounded edges is considered to be negligible to the dose rate along the bisector axis perpendicular to the long axis of the source. A density of 22.42 g cm^–3^ is used for the core. The radioactivity is uniformly distributed within the metal source. A stainless steel shell encapsulates the source. The capsule is a cylinder of 0.9 mm outer diameter, 0.7 mm inner diameter, and 4.5 mm length. An air gap of 0.1 mm thickness exists between the metallic core and the capsule. One end of the capsule is rounded with a half‐sphere of radius 0.45 mm. The other end is beveled and welded to a stainless steel cable. The beveled end of the capsule is modeled with a cone. The cable is a cylinder of 0.7 mm diameter and 200 mm length. Only 7.5 mm of cable is modeled in this study. The stainless steel used in all components is AISI 316L steel of 8.02 g cm^–3^ density with the following elemental composition, by weight: 2% Mn, 1% Si, 17% Cr, 12% Ni, and 68% Fe.

The photon energy spectrum of the simulated I192r source was taken from the U.S. Department of Energy Radioactive Decay Tables.^(10)^ The photon energy spectrum includes 26 energies with 2.36 particles per disintegration. The energy spectrum ranges from 8.91 keV to 871.73 keV. Beta particles emitted from the source were not included because they will not contribute to dose outside the source due to their short ranges. The photon emission from the cylindrical source is assumed to be isotropic.

**Figure 1:**
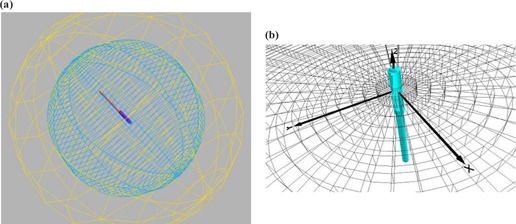
Monte Carlo model phantom geometry. (a) The HDR source is centered inside a spherical inner balloon with a concentric outer sphere, which represents a patient's tissue. (b) The tallying grid consists of concentric cylinders of increasing radii centered at the core of the source.

The software used to perform the simulation was the Monte Carlo *N*‐particle transport code MCNPX (v2.4.0; Los Alamos National Laboratory, Los Alamos, NM).^(11)^ The code was used to calculate the energy deposited per unit volume in the phantom. To yield fine resolution in the calculations, this simulation used a cylindrical tallying grid in increments of 1 mm along the radial axis (the difference between the radii of consecutive concentric cylinders) and width of 0.1 mm wide (the cylinder height) centered at the core of the source. The tallying grid covered distances from the balloon's surface to the end of the phantom. The phantom and the tallying grid are shown in Fig. 1.

Cross‐sectional data for all photon and electron interactions were taken from the Evaluated Nuclear Data File, vB‐VI,^(11)^ and the MCNPX simulation accounted for Compton scattering, the photoelectric effect, and coherent scattering. Characteristic X‐rays produced by photoelectric absorption were also included in the calculations. The photon cutoff energy was set at 2 keV, which is adequate for the present grid resolution. Because of the low energies of photons emitted by the I192r source, secondary charged‐particle equilibrium can be assumed to exist, and the absorbed dose can therefore be approximated by collision kerma calculations.^(12)^ To reduce the uncertainty of the Monte Carlo calculations to less than 0.5%, histories of 10 000 000 photons were tracked for each MCNPX simulation.

Dose rates were normalized to air kerma strength. The air kerma strength was calculated by MCNPX using a separate simulation in which the source was centered in a sphere of 5 m diameter with composition of dry air. The air kerma strength was calculated 1 m from the center of the source. The cylindrical grid used for this calculation had a larger scoring bin width of 1 cm and a step of 0.6 cm in order to achieve adequate uncertainty in the Monte Carlo calculations.

## III. RESULTS AND DISCUSSION

The Monte Carlo model was validated against the dosimetry characterization work of the same HDR I192r source performed by Daskalov et al. (1998).^(9)^ The dose rates at various distances from the center of the source (along the bisector of the source) in water were compared. Daskalov et al. used a different Monte Carlo computer code and energy spectrum. The source geometry used by Daskalov et al. is the same as that applied here with slight differences in the modeling of the beveled edges of the core and capsule. However, these minor differences have negligible contribution to the present calculations performed away from the source along the bisector axis. The dose rate constant (defined as the dose rate per unit air kerma strength at 1 cm in water) obtained in the present calculations is $1.107\pm 0.22\%\;\text{cGy}\;h^{-1}U^{-1}$ while that reported by Daskalov et al. is $1.108\pm 0.13\%\;\text{cGy}\;h^{-1}U^{-1}$. This is also in agreement with their thermoluminescent dosimeter measurement of the dose rate constant, which varied from 1.10 to $1.12\;\text{cGy}\;h^{-1}U^{-1}$. Corresponding dose rates reported by Daskalov et al. at distances of 1.5 cm, 2 cm, 2.5 cm, 3 cm, and 5 cm were all within 0.6% of the present calculations. The air kerma strength 1 m from the center of the source obtained in the present calculation and used to normalize the dose rates was 3.624 ± 0.16% U per mCi (0.098 U per MBq) of contained activity.

**Figure 2:**
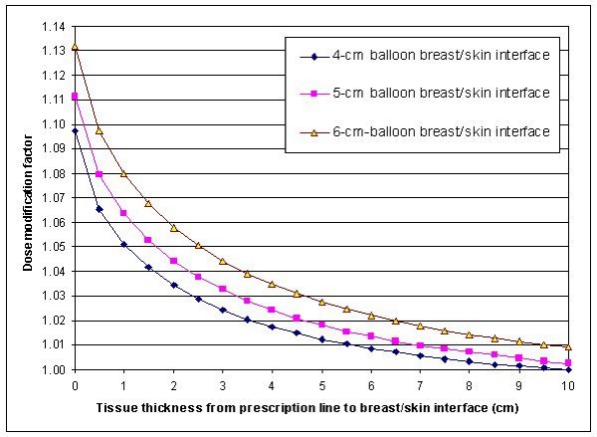
Dose modification factors at the prescription line 1 cm from the balloon's surface with various breast tissue thicknesses between the prescription line and the breast–skin interface

The dose modification factors at the prescription line 1 cm from the balloon's surface near the breast–skin interface for balloon diameters of 4 cm, 5 cm, and 6 cm and tissue thicknesses ranging from 0 cm to 10 cm beyond the prescription line are plotted in Fig. 2. In clinical applications, it would be rare to have much tissue beyond about 5 cm from the prescription line. However, the range was extended to 10 cm to better illustrate the amount of tissue needed for full scatter and for the sake of completeness. The dose modification factors for the breast–skin interface when there was no tissue beyond the prescription line (i.e., only 1 cm of breast tissue existed between the balloon's surface and the skin) were 1.098, 1.112, and 1.132 for the 4‐cm, 5‐cm, and 6‐cm balloons, respectively. Therefore, even when the procedure criterion for a minimum of 1 cm between the balloon's surface and the skin was met, the reduction in the dose rate resulting from the lack of full scatter was approximately 10% (range, 9% to 12%, depending on the balloon's diameter). Furthermore, the simulations showed that 5 cm of breast tissue beyond the prescription line (6 cm of tissue between the balloon's surface and the skin) could not result in full scatter, with dose modification factors ranging from 1.012 for the smallest balloon to 1.028 for the largest. Ye et al.^(^ ^13^ ^,^ ^14^ ^)^ reported similar results, with a 3% to 9% dose rate reduction at the prescription line in Monte Carlo simulations of a balloon with a diameter of 4.5 cm, depending on the amount of tissue between the balloon's surface and the skin.

**Figure 3:**
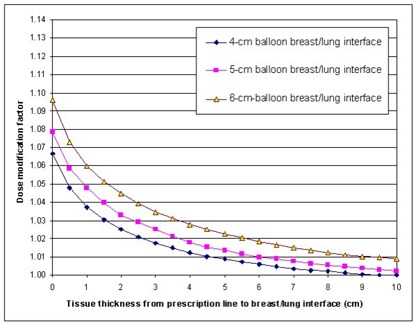
Dose modification factors at the prescription line 1 cm from the balloon's surface with various breast tissue thicknesses between the prescription line and the breast–lung interface

The dose modification factors at the prescription line 1 cm from the balloon's surface near the breast–lung interface for balloon diameters of 4 cm, 5 cm, and 6 cm and breast tissue thicknesses ranging from 0 cm to 10 cm beyond the prescription line are plotted in Fig. 3. The factors for the breast–lung interface were lower than those for the breast–skin interface. This is, of course, because lung tissue is so much denser than air and thus provides more scatter than air does. The dose modification factor for the breast–lung interface ranged from 1.067 when there was no additional breast tissue beyond the prescription line to 1.009 when there was 5 cm of tissue beyond the prescription line for the 4‐cm balloon. For the largest balloon, the dose modification factors were 1.096 and 1.023 with 0 cm and 5 cm of breast tissue beyond the prescription line, respectively. Thus, the reduction in the dose rate resulting from the lack of full scatter was approximately as high as 6% to 9%, depending on the balloon's diameter. In addition, as in the case of the breast–skin interface, even with 5 cm of breast tissue beyond the prescription line, full scatter did not exist.

Although it is generally not strictly followed, the criterion for the minimum distance from the balloon's surface to the lung is typically the same as for the distance from the balloon's surface to the skin. Therefore, even if this criterion is met, the reduction in the dose resulting from the lack of scatter can still be considerable. It can be argued that the bone in the chest wall might provide additional scatter, so the scatter effect at the breast–lung interface therefore might not be as pronounced as our simulations indicated. However, the presence of bone in the chest wall would affect the dose rate only over a limited region, so it was not modeled in this study.

It is possible to compensate for a lack of tissue beyond the prescription line by adding bolus. The present results showed that the effect of added bolus was appreciable with the first 2 cm of thickness but diminished beyond 5 cm. Some clinical trials have shown adverse effects on the skin.^(^ ^1^ ^–^ ^3^ ^,^ ^15^ ^)^ These adverse effects may actually be caused at lower doses than previously reported when heterogeneity is accounted for and the lack of full scatter is considered.

The present modeling assumes full symmetry with air or lung surrounding the entire 3D geometry. Although this procedure typically is treated with balloon–source symmetry of ±2 mm along the orthogonal bisector plane of the source, various amounts of tissue would be present at different angles in the clinical cases rather than a constant tissue thickness at all angles, as the present simulations were performed. By averaging the factors at various tissue thicknesses over multiple angular sectors, one can obtain an average dose modification factor for the implant. In addition, dose optimization techniques can be developed with multiple dwell positions to account for this dose modification.

Because the Association of Physicists in Medicine Task Group 40 recommendation for intracavitary brachytherapy allows for ±15% in the delivery of the prescribed dose rather than the ±5% limit expected for external beam therapy,^(16)^ the reduction in the dose resulting from the lack of full scatter coupled with the reduction from the use of high atomic number contrast material (which causes a dose rate reduction in the range of 1% to 6%)^(^ ^6^ ^,^ ^17^ ^,^ ^18^ ^)^ can result in considerable uncertainty. A comparison of the dosimetry with and without a correction for heterogeneity may, therefore, be helpful, especially in cases in which there is not much tissue beyond the prescription line and the procedure uses a large balloon with a highly concentrated contrast material. One should keep in mind that most breast tumor recurrences are in or near the original tumor bed,^(19)^ which is the region this procedure is intended to cover. Therefore, adequate coverage is essential, and it is important to consider the effect of diminished scatter to achieve adequate dosimetry.

## IV. CONCLUSION

Our Monte Carlo simulations showed dose rate reductions of 9% for the smallest balloon and 12% for the largest balloon when the criterion for the minimum distance between the balloon's surface and the skin was met. The dose reduction at the breast–lung interface was 6% for the smallest balloon and 9% for the largest. The dose reduction, therefore, warrants comparing plans with a correction for heterogeneity and plans that assume homogeneity. Not accounting for the dose modification factors may result in delivery of a lower dose than is prescribed. Our findings also show the importance of ensuring the required minimum distance between the balloon's surface and the breast's interfaces with both the lung and skin for accurate dosimetry in this brachytherapy procedure.

## Supporting information

Supplementary Material
